# Quantitative proteomic analysis reveals the influence of plantaricin BM-1 on metabolic pathways and peptidoglycan synthesis in *Escherichia coli* K12

**DOI:** 10.1371/journal.pone.0231975

**Published:** 2020-04-23

**Authors:** Huan Wang, Yuanhong Xie, Hanwei Zhang, Junhua Jin, Hongxing Zhang

**Affiliations:** Beijing Laboratory of Food Quality and Safety, Beijing Key Laboratory of Agricultural Product Detection and Control of Spoilage Organisms and Pesticide Residue, College of Food Science and Engineering, Beijing University of Agriculture, Beijing, China; Roskilde Universitet, DENMARK

## Abstract

Plantaricin BM-1 is a class IIa bacteriocin with a strong bactericidal effect on gram-positive bacteria. Although plantaricin BM-1 also inhibits the growth of some gram-negative bacteria, including *Escherichia coli*, the mechanism is not clear. In this study, we used tandem mass tag-based quantitative proteomics analysis to examine the inhibitory mechanism of plantaricin BM-1 against *E*. *coli* K12, and evaluated the morphological effects by electron microscopy. The results demonstrated that plantaricin BM-1 inhibits the growth of *E*. *coli* K12 by bacteriostatic action, mainly acting on the surface of the cell wall, leading to its collapse. Proteomic analysis identified 976 differentially expressed proteins (>1.2-fold change, *p* < 0.05) under treatment with plantaricin BM-1, including 490 up-regulated proteins and 486 down-regulated proteins. These proteins were mainly involved in peptidoglycan synthesis and energy metabolism pathways, including amino acid, glyoxylate and dicarboxylate, ABC transporter, and quorum-sensing pathways. Specifically, plantaricin BM-1 treatment significantly improved peptidoglycan synthesis and enhanced the tricarboxylic acid cycle in *E*. *coli* K12, and altered the expression of cell membrane proteins. These results provide new insight into the inhibition mechanism of class IIa bacteriocins on gram-negative bacteria, which can lay the foundation for its broader use as an alternative to conventional antibiotics.

## Introduction

With the continuous emergence of antimicrobial-resistant bacterial strains, representing a serious clinical and public health challenge, antibiotic alternatives are needed [1, 2]. In particular, bacteriocins and other antimicrobial peptides should be considered as replacements for traditional antibiotics [3]. Bacteriocins are ribosomally synthesized antimicrobial peptides that show high specificity against closely related bacteria [4]. Bacteriocins can be divided into four distinct classes according to their chemical structure, stability, and molecular weight [5]: (I) lantibiotics, which are small (<5 kDa) membrane-active peptides; (II) small (<10 kDa), heat-stable, non-lanthionine peptides; (III) large (>10 kDa) heat-labile proteins; and (IV) complex bacteriocins [6]. Among them, Nisin of class I and IIa of class II bacteriocins being the most abundant and thoroughly studied [7]. Nisin is the only bacteriocin approved by FDA to be used as food preservative [6]. The application of class IIa bacteriocin in food preservation is still in the experimental stage, but it has application prospects in many aspects [7]. The main mechanism of action of class IIa bacteriocins is by increasing the permeability of the target cell membrane, including through the formation of ion leakage pores that leads to a proton-motive force and ATP dissipation [8]. The IICD component of the mannose phosphotransferase system (man-PTS) of susceptible cells is considered to serve as the main receptor for class IIa bacteriocins, leading to bacterial death [9–11]; however, this is not the target of class IIa bacteriocins in gram-negative bacteria [12].

The general insensitivity of gram-negative bacteria to bacteriocins is considered to be related to the existence of lipopolysaccharide and the outer membrane that prevent contact between the bacteriocin and the cell membrane [13]. In addition, two Tat-dependent peptidoglycan amidases that regulate the peptidoglycan levels between the outer and inner membranes were reported to confer resistance of gram-negative bacteria to antimicrobial peptides [14].

In contrast to this general pattern, plantaricin BM-1 is a class IIa bacteriocin that shows the ability to inhibit both gram-positive and gram-negative bacteria. Plantaricin BM-1 is produced by *Lactobacillus plantarum* BM-1 isolated from Chinese traditional fermented meat products, and shows a strong bactericidal effect against *Listeria monocytogenes* but was also shown to inhibit the growth of *Escherichia coli* [15, 16]. However, the precise mechanism of this inhibition has not yet been elucidated.

In this study, we investigated the inhibition effect of plantaricin BM-1 on *E*. *coli* K12. We used a proteomics approach and electron microscope observations to determine the changes to the cell morphology, proteins, and related pathways of *E*. *coli* K12 treated with plantaricin BM-1. These findings can provide insight into the mechanism of action of plantaricin BM-1 against gram-negative bacteria as a candidate replacement for conventional antibiotics.

## Materials and methods

### Bacterial strains and growth conditions

*E*. *coli* K12 BW25113 was cultured in Luria-Bertani (LB) broth at 37°C. To obtain plantaricin BM-1, *Lactobacillus plantarum* BM-1 was cultured in de Man, Rogosa and Sharpe (MRS) broth at 37°C. All strains were stored in 15% (v/v) glycerol at –80°C.

### Bacteriocin preparation

Purification of plantaricin BM-1 was as described by Zhang et al [15]. Briefly, *Lactobacillus plantarum* BM-1 was cultured in MRS broth at 37°C for 20 h. The supernatant fermentation broth was collected by centrifugation, and plantaricin BM-1 was purified using dialysis desalting and cation exchange. The purified plantaricin BM-1 was sterilized through a 0.22-μm Millex GP filter, and then lyophilized and re-dissolved with sterile water. The bacteriocin titer was determined by the agar well diffusion method [15] and expressed in arbitrary units (AU) per milliliter.

### Growth curve of *E*. *coli* K12 under treatment with plantaricin BM-1

To determine the growth inhibitory effect, *E*. *coli* K12 was cultured in LB broth at a final concentration of 4.5 × 10^4^ colony-forming units (CFU)/mL without or with plantaricin BM-1 (512 AU/mL) at 37°C for 12 h. The optical density at 600 nm (OD_600nm_) and the viable count of *E*. *coli* K12 were measured every 2 h. All experiments were repeated three times.

### Morphological analysis

*E*. *coli* K12 was cultured in LB broth (final concentration of 4.5 × 10^4^ CFU/mL) without or with plantaricin BM-1 (512 AU/mL) at 37°C for 12 h. The cells were centrifuged (8000 rpm, 10 min at 4°C) and collected for scanning electron microscopy (SEM) and transmission electron microscopy (TEM).

For SEM, the cells were fixed with 2.5% glutaraldehyde for 2 h at room temperature and washed with phosphate-buffered saline (0.1 mol/L) three times. Dehydration was performed sequentially with 30%, 50%, 70%, 80%, and 90% ethanol for 10 min each, followed by treatment with 100% ethanol for 20 min and 100% tert-Butanol for 20 min; all steps were repeated three times [17, 18]. After freeze-drying, each sample was plated with ion-splashing gold and observed with SEM (SU8010, Hitachi, Japan).

For TEM, *E*. *coli* K12 cells were treated in the same manner as described above for SEM until the ethanol dehydration steps. After dehydration, the cells were permeated in acetone (2:1) as the embedding agent at 35°C for 2 h and then polymerized at 65°C for 48 h. Ultrathin sections (approximately 50 nm) were prepared on copper grids and stained with 2% uranyl acetate and lead citrate [19], and then observed with TEM (HT7800, Hitachi, Japan).

### Protein preparation and tandem mass tag (TMT) labeling

*E*. *coli* K12 was cultured in LB broth (final concentration of 4.5 × 10^4^ CFU/mL) without or with plantaricin BM-1 (512 AU/mL) at 37°C for 12 h. After centrifugation, the supernatant was collected and treated with protein lysis buffer (8 M urea + 1% sodium dodecyl sulfate, protease inhibitor) to obtain the total protein. The concentration of the extracted protein was determined by the Pierce BCA Protein Assay Kit (Thermo Fisher). Demethylation was performed by adding 10 mM Tris (2-carboxyethyl) phosphine and 40 mM iodoacetamide into the total protein, and then the proteins were hydrolyzed by trypsin at a 1:50 ratio. The hydrolytic peptides were labeled with a TMT labeling kit (Thermo Fisher) and then subjected to liquid chromatography-tandem mass spectrometry (LC-MS/MS). Three biological replicates were prepared for each sample.

### Protein identification and quantification

High-pH liquid-phase separation of TMT-labeled peptides was performed using a reversed-phase liquid chromatography system (Thermo Scientific Vanquish Flex, Thermo Scientific) equipped with a reversed-phase C18 column (ACQUITY UPLC BEH C18 Column 1.7 μm, 2.1 mm × 150 mm, Waters, USA). The peptide elution was monitored at 214 nm. After 5 min, the eluted peptides were collected every minute, pooled into 10 fractions, and then lyophilized.

Dried fractions obtained from the high-pH reverse-phase separations were run on a Q Exactive mass spectrometer (Thermo Scientific) with a C18 column (75 μm × 25 cm, Thermo Scientific) for identification and quantification using previously reported identification parameters [20].

LC-MS/MS data were matched using Proteome Discoverer ^TM^ Software 2.2, and searched using the uniprot-*Escherichia coli* (strain K12) [83333]-4353s-20190412 database with a peptide false discovery rate ≤ 0.01. Only proteins involving at least one unique peptide were used for quantification. Subsequently, the differentially expressed proteins were identified according to a fold-change > 1.2 or < 0.83 between treatments and *p* < 0.05. Kyoto Encyclopedia of Genes and Genomes (KEGG) pathway enrichment analysis (http://www.genome.jp/kegg/) was used to identify the functional subclasses and metabolic pathways of the differentially expressed proteins.

### Statistical analyses

All experiments were performed in triplicate, and data are presented as the mean ± SD. Analysis of variance was used for comparison of mean values of the OD_600nm_ and viable count of *E*. *coli* K12 and *E*. *coli* K12 treated with plantaricin BM-1 at a significance level of 0.05.

## Results and discussion

### Effect of plantaricin BM-1 on the growth of *E*. *coli* K12

Class IIa bacteriocins have strong antibacterial activity against a variety of gram-positive bacteria, but are generally not effective against gram-negative bacteria [13]. In line with this general finding, we previously showed that *L*. *plantarum* BM-1 produces a class IIa bacteriocin, plantaricin BM-1, with a strong bactericidal effect on some gram-positive bacteria, including *L*. *monocytogenes*. Moreover, analysis of the antimicrobial spectrum indicated that plantaricin BM-1 could also inhibit the growth of some gram-negative bacteria, including *E*. *coli* [15]. Therefore, in the present study we sought to uncover the mechanism of this inhibition.

**Fig 1:**
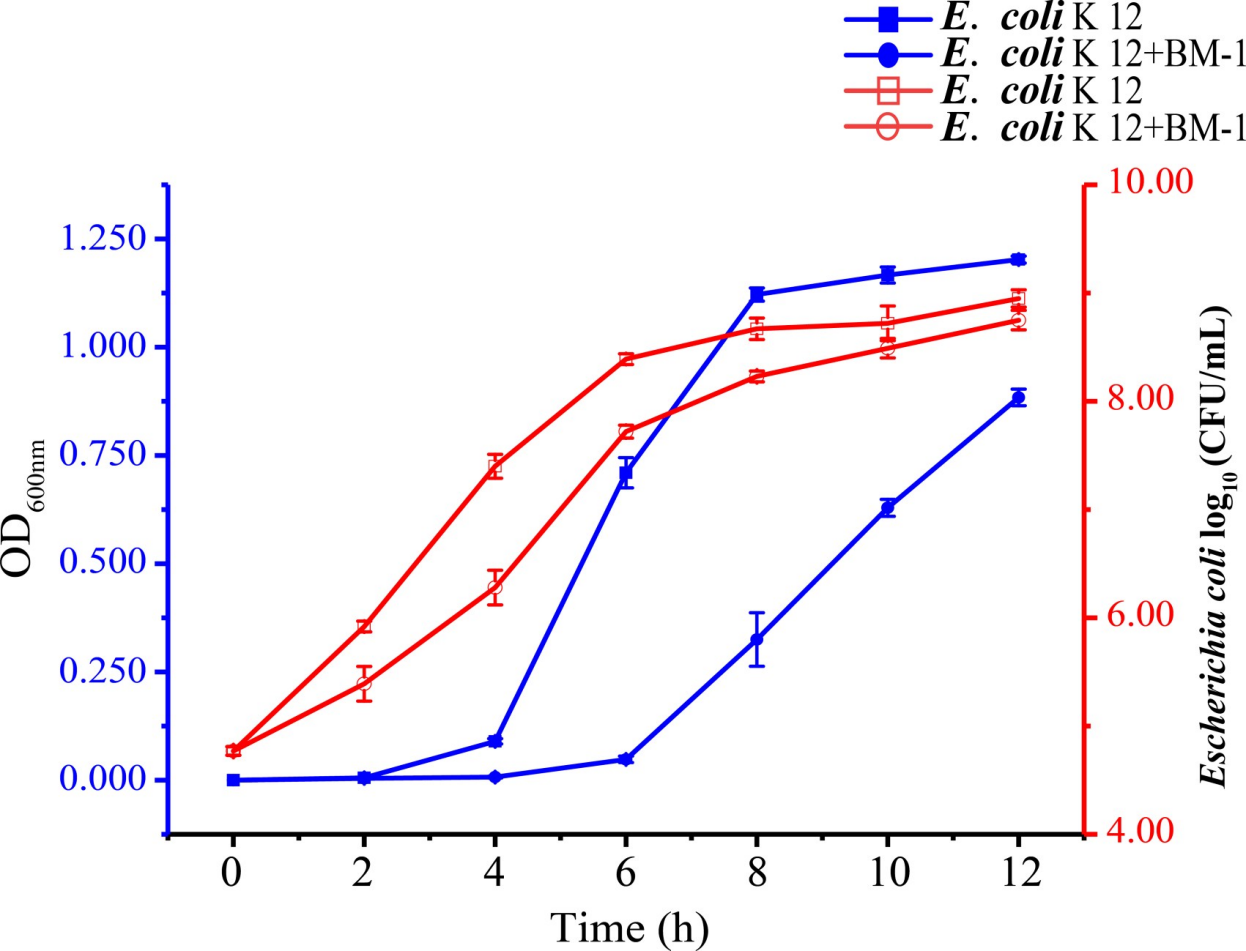
Growth changes of *E*. *coli* K12 treated with plantaricin BM-1 (512 AU/mL). The blue line represents the growth curve and the red line indicates the number of living bacteria.

When grown in liquid LB medium, the OD_600nm_ of *E*. *coli* K12 increased gradually from 0 h to 4 h, and then increased sharply to 1.202 at 12 h. Under treatment with plantaricin BM-1 (512 AU/mL), the OD_600nm_ of *E*. *coli* K12 was stable from 0 h to 6 h, and then increased dramatically to 0.884 at 12 h, representing a significantly lower level than that of the control at the same time point (Fig 1). Similarly, the viable count of *E*. *coli* K12 increased gradually throughout the 12 h culture period, but was always significantly lower than that of the control group. These results indicated that plantaricin BM-1 could in fact inhibit the growth of *E*. *coli* K12 via bacteriostatic action rather than bactericidal action. This result is in line with a previous study showing that class IIa bacteriocins only have obvious bactericidal effects on gram-positive bacteria [12].

### Morphological changes of *E*. *coli* K12 under plantaricin BM-1

**Fig 2:**
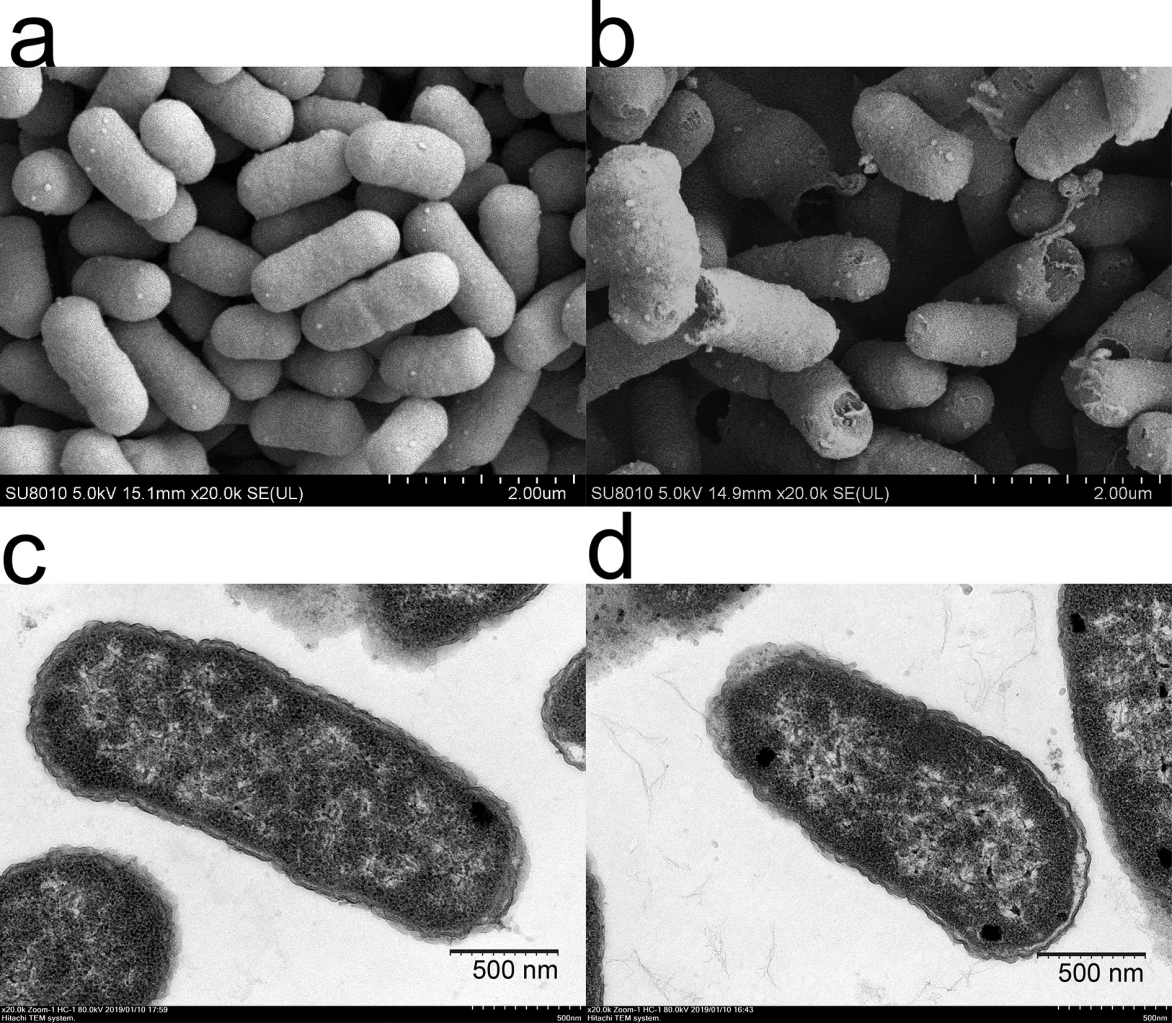
Electron microscopy observations of *E*. *coli* K12 with and without (control) plantaricin BM-1 (512 AU/mL) treatment. (a) SEM of control; (b) SEM of treated cells (magnification ×20,000); (c) TEM of control; (d) TEM of treated cells (magnification ×20,000).

The SEM images was shown in Fig 2, the control *E*. *coli* K12 cells had a relatively intact cell morphology with a smooth surface and plump cell profile, and the edges were neat and complete. However, addition of plantaricin BM-1 to the culture medium resulted in contracted and ruptured cells, and the cell surface was collapsed at either end with obvious debris detectable around the cell. TEM images showed that in the absence of plantaricin BM-1, the cell wall was intact and rod-shaped, the edges of the cells were smooth, and the cytoplasm was evenly distributed (Fig 2C). Under treatment with plantaricin BM-1, the bacteria cells largely maintained their normal rod shape; although the cytoplasm of some bacteria was contracted, most bacteria showed a normal morphology. However, the blurring of the periplasmic space of the contracted cells was not as visible as that in the normal cells (Fig 2D). These results indicated that the peptidoglycan and phospholipid layer of the cell wall had been affected by plantaricin BM-1. Thus, plantaricin BM-1 appears to inhibit the growth of *E*. *coli* K12 by acting on the cell surface to prevent normal cell differentiation. At present, it is generally believed that bacteriocin damages or kills target cells by forming pores in the cell membrane [3, 11]. Bacteriocin is recognized by outer membrane proteins and then passes through the membrane through a mechanism dependent on the outer membrane receptor TonB, eventually forming pores in the inner membrane [3]. We believe that phytolactam BM-1 inhibits the growth of *E*. *coli* K12 due to the rupture and depression of the cell membrane.

### Proteome and KEGG pathway analysis

To further understand the strategy by which *E*. *coli* K12 copes with bacteriocins stress, we used a proteomics approach with TMT labeling. Based on this strategy, a total of 2206 proteins were identified, including 976 proteins that were differentially expressed with a greater than 1.2-fold change (*p* < 0.05) in *E*. *coli* K12 treated with plantaricin BM-1. Among these, there were 490 up-regulated and 486 down-regulated proteins.

In particular, the expression level of glucose phosphate isomerase Gpi (P0A6T1), which catalyzes the conversion between glucose-6-phosphate (Glc-6p) and fructose-6-phosphate (Fru-6p), was up-regulated by 1.39-fold under plantaricin BM-1 treatment. Moreover, the expression of d-fructose-6-phosphate aminotransferase GlmS (P17169), which can convert Fru-6p to glucosamine-6-phosphate (GlcN-6p), was also up-regulated by 1.27-fold. Along with GlcN-6p, nine other differentially regulated proteins are involved in the synthesis of peptidoglycan, all of which were up-regulated (Table 1). This result is in line with the SEM and TEM observations, indicating that *E*. *coli* K12 can repair the cellular damage caused by plantaricin BM-1 by increasing the synthesis of peptidoglycan. A previous study demonstrated that nisin, interacts with the peptidoglycan precursor lipid II of gram-positive bacteria, which was proposed to act as a docking molecule [21]. Thus, nisin has a dual function since its binding to lipid II inhibits peptidoglycan biosynthesis [22]. Gravesen (2001) suggested that the mechanism contributing to increased nisin resistance involves changes in the cell envelope that reduce the access to or sensitivity of the membrane [23]. Moreover, alteration of the expression of *pbp2A* in *L*. *monocytogenes* affected the synthesis of peptidoglycan, as the main component of the cell wall, thereby preventing the binding of nisin to lipid II, which resulted in resistance to nisin [23]. Unlike gram-positive bacteria, the peptidoglycan of *E coli* is not located outside the outer membrane, but between the outer and the inner membrane [24]. Moreover, no studies have clearly shown that IIa bacteriocins directly act on peptidoglycans. Therefore, results suggested that *E*. *coli* K12 copes with the stress of plantaricin BM-1 by increasing the synthesis of peptidoglycan.

**Table 1:** Up-regulated proteins in peptidoglycan synthesis.

Accession	Description	Fold change	*P* value	Protein	Function
P0A6T1	Glucosephosphate isomerase	1.39	0.000115	Gpi	窗体顶端
					Enzyme; Energy metabolism, carbon: Glycolysis
P17169	窗体顶端	1.27	0.002353	GlmS	窗体顶端
	Glutamine:D-fructose-6-phosphate aminotransferase				Enzyme; Central intermediary metabolism:Amino sugars
P0AFI5	Alanyl-D-alanine endopeptidase	1.74	0.000651	PbpG	Putative enzyme; murein sacculus, peptidoglycan
P33013	alanyl-D-alanine carboxypeptidase	1.58	0.005223	DacD	Putative enzyme; murein sacculus, Peptidoglycan
P0A6J8	Alanine-D-alanine ligase A	1.42	0.000187	DdlA	Enzyme; murein sacculus, peptidoglycan
P0AF16	Putative peptidoglycan lipid II flippase	1.41	0.006233	MurJ	Putative factor; not classified
P0AEB2	Alanyl-D-alanine carboxypeptidase	1.40	0.000244	DacA	Enzyme; murein sacculus, peptidoglycan
P17952	UDP-N-acetylmuramate: L-alanine ligase	1.29	0.002586	MurC	Enzyme; murein sacculus, peptidoglycan
P22634	Glutamate racemase	1.28	0.001415	MurI	Enzyme; murein sacculus, peptidoglycan
P11880	UDP-N-acetylmuramoyl-tripeptide: D-alanyl-D-alanine ligase	1.26	0.001793	MurF	Enzyme; murein sacculus, peptidoglycan
P0AD65	Transpeptidase involved in peptidoglycan synthesis	1.23	0.02935	MrdA	Enzyme; cell division

**Fig 3:**
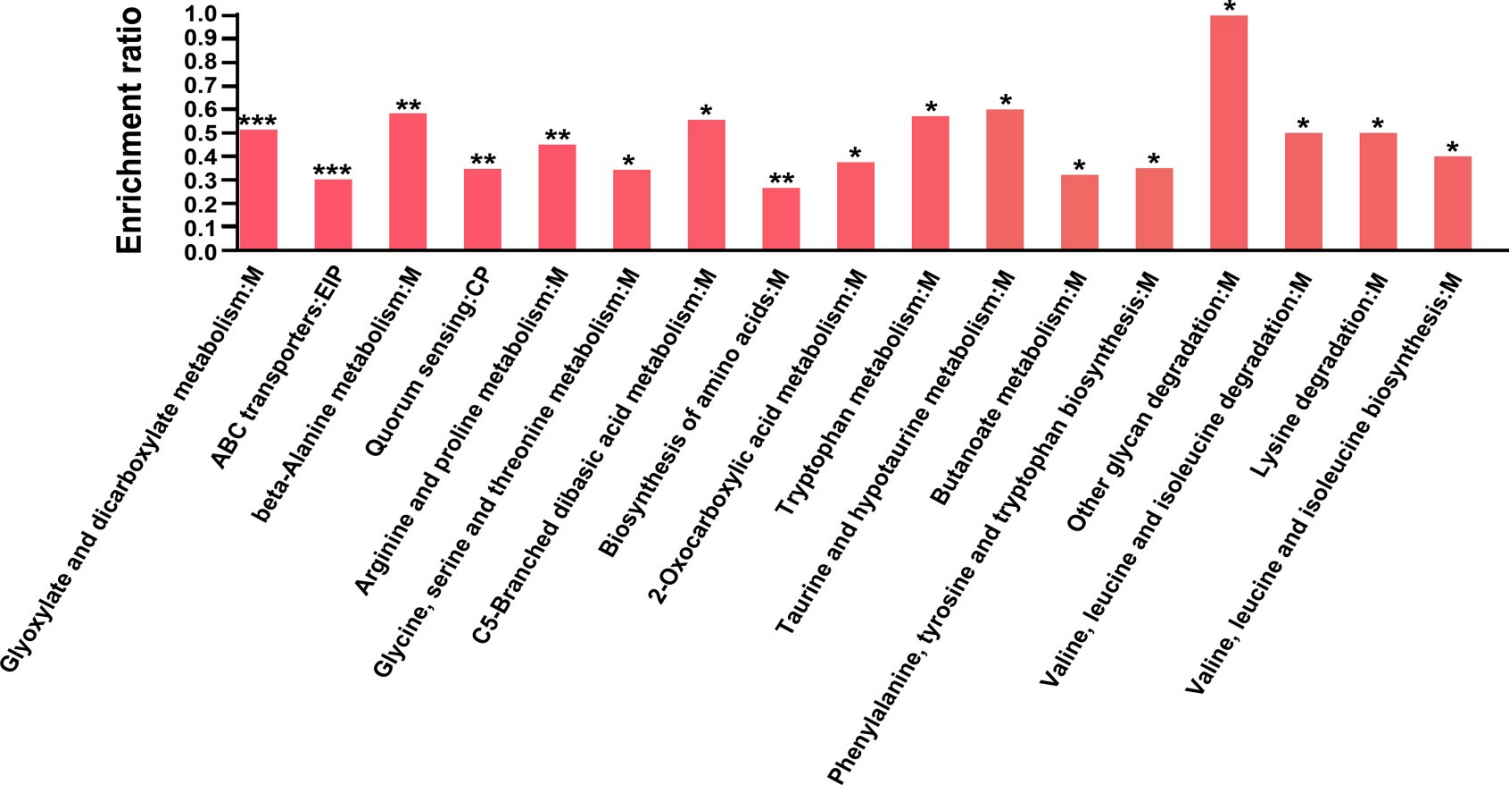
KEGG pathway enrichment analysis of proteins down-regulated in response to plantaricin BM-1 treatment. *p < 0.05, **p < 0.01, ***p < 0.001.

**Fig 4:**
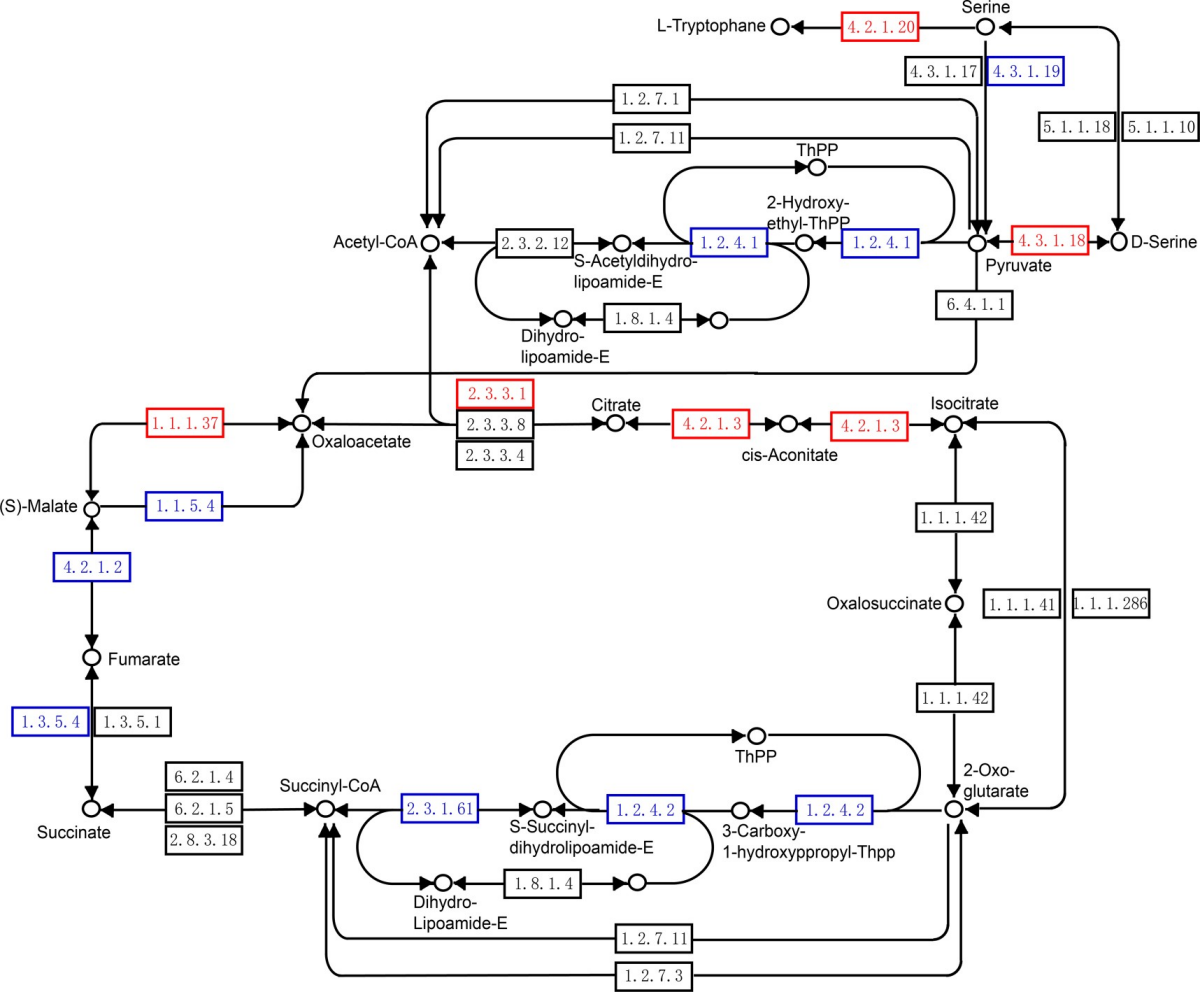
Changes in proteins involved in the tricarboxylic acid (TCA) cycle of *E*. *coli* K12 after treatment with plantaricin BM-1. Red indicates down-regulated proteins and blue indicates up-regulated proteins.

KEGG pathway analysis showed significant (*p* < 0.05) enrichment of the proteins with down-regulated expression in metabolic pathways, suggesting an important role of metabolism in the response to plantaricin BM-1 (Fig 3). Specifically, pathways involved in the metabolism of amino acids (valine, leucine, and isoleucine), glyoxylate and dicarboxylate, and butanoate were identified to be down-regulated, which either directly or indirectly lead to reduced acetyl coenzyme A (acetyl-CoA) synthesis. Surprisingly, tryptophan synthase alpha subunit TrpA (P0A877) and beta subunit TrpB (P0A879), which are both involved in serine metabolism, were down-regulated, thereby reducing l-tryptophan biosynthesis. Previous studies have shown that amino acid metabolism is directly involved in Nisin resistance [25], and serine biosynthesis is also related to *E*. *coli* resistance [26]. In addition, l-threonine dehydratase TdcB (P0AGF6), which regulates the synthesis of pyruvate from serine, was up-regulated by 2.90-fold. Further conversion of pyruvate to acetyl-CoA accelerates the tricarboxylic acid cycle (TCA cycle) (Fig 4 and Table 2). This pathway represents a rare strategy to accelerate the synthesis of acetyl-CoA in the presence of plantaricin BM-1. GlcN-6p is converted to N-acetylglucosamine-6-phosphate (GlcNAc-6p) under catalysis by acetyl-CoA, which accelerates the synthesis of peptidoglycan. Moreover, the TCA cycle can provide a large amount of ATP for the conversion of glucose into Glc-6p at the beginning of peptidoglycan synthesis. Therefore, both the increase in the synthesis of acetyl-CoA and the large amount of ATP produced by the accelerated TCA cycle caused by acetyl-CoA play an important role in the synthesis of peptidoglycan. In our study, the TCA cycle was accelerated after 12 h of treatment with plantaricin BM-1 in *E*. *coli* K12, which is in contrast to the results of Miao et al. (2015), who showed that treatment with the novel bacteriocin peptide F1 (molecular weight of approximately 2 kDa) for only 30 min inhibited the growth of *E*. *coli* [27]. In addition, they found that F1 down-regulated the fumarate hydratase FumB, which inhibited the TCA cycle, whereas the ClpP-ClpX protease system was activated. By contrast, in the present study, FumB expression (P14407) was up-regulated by 1.60-fold after treatment with plantaricin BM-1 (Table 2), and the ClpP-ClpX protease system did not change significantly. We speculate that the reason for this difference is mainly due to the different treatment times of the bacteriocins on *E*. *coli*. In addition, the different types of bacteriocins may also have distinct impacts on metabolic pathways in *E*. *coli*.

**Table 2:** Differentially regulated proteins in the tricarboxylic acid cycle of *E*. *coli* K12 under plantaricin BM-1.

Accession	Description	Fold change	*P* value	Protein	Function
P0AGF6	窗体顶端 threonine dehydratase, catabolic	2.90	0.00001	TdcB	窗体顶端
					Enzyme; Degradation of small molecules: Amino acids
P0A877	窗体顶端	-1.71	0.000148	TrpA	窗体顶端
	Tryptophan synthase, alpha subunit				Enzyme; Amino acid biosynthesis: Tryptophan
P0A879	窗体顶端	-1.47	0.000148	TraB	窗体顶端 Enzyme; Amino acid biosynthesis: Tryptophan
	trypt窗体顶端				
	Tryptophan synthase, beta subunit				
	窗体底端				
	ophan synthase, beta subunit				
P0AFG8	窗体顶端	1.24	0.004198	AceE	窗体顶端 Enzyme; Energy metabolism, carbon: Pyruvate dehydrogenase
	Pyruvate dehydrogenase, decarboxylase component E1, thiamine triphosphate-binding				
P0ABH7	窗体顶端 Citrate synthase	-1.33	0.000768	GltA	窗体顶端
					Enzyme; Energy metabolism, carbon: TCA cycle
P61889	窗体顶端	-1.24	0.000474	Mdh	窗体顶端
	Malate dehydrogenase, NAD(P)-binding				Enzyme; Energy metabolism, carbon: TCA cycle
P25516	窗体顶端	-2.10	0.000075	AcnA	窗体顶端
	Aconitate hydratase 1				Enzyme; Energy metabolism, carbon: TCA cycle
P33940	窗体顶端	1.80	0.001449	Mqo	Unknown
	Malate dehydrogenase, FAD/NAD(P)-binding domain				
P14407	窗体顶端	1.60	0.000371	FumB	窗体顶端
	Anaerobic class I fumarate hydratase				Enzyme; Energy metabolism, carbon: TCA cycle
P00363	窗体顶端	1.40	0.000942	FrdA	窗体顶端
	Fumarate reductase (anaerobic) catalytic and NAD/flavoprotein subunit				Enzyme; Energy metabolism,
					carbon: Anaerobic Respiration
P0AC47	窗体顶端	1.26	0.006763	FrdB	窗体顶端
	Fumarate reductase (anaerobic), Fe-S subunit				Enzyme; Energy metabolism, carbon: Anaerobic respiration
P0AFG6	窗体顶端	1.40	0.0003	SucB	窗体顶端
	Dihydrolipoyltranssuccinase				Enzyme; Energy metabolism, carbon: TCA cycle
P0AFG3	窗体顶端	1.47	0.001002	SucA	Enzyme; Energy metabolism, carbon: TCA cycle
	2-oxoglutarate decarboxylase,thiamine triphosphate-binding				

### Effect of plantaricin BM-1 on *E*. *coli* K12 membrane proteins

**Fig 5:**
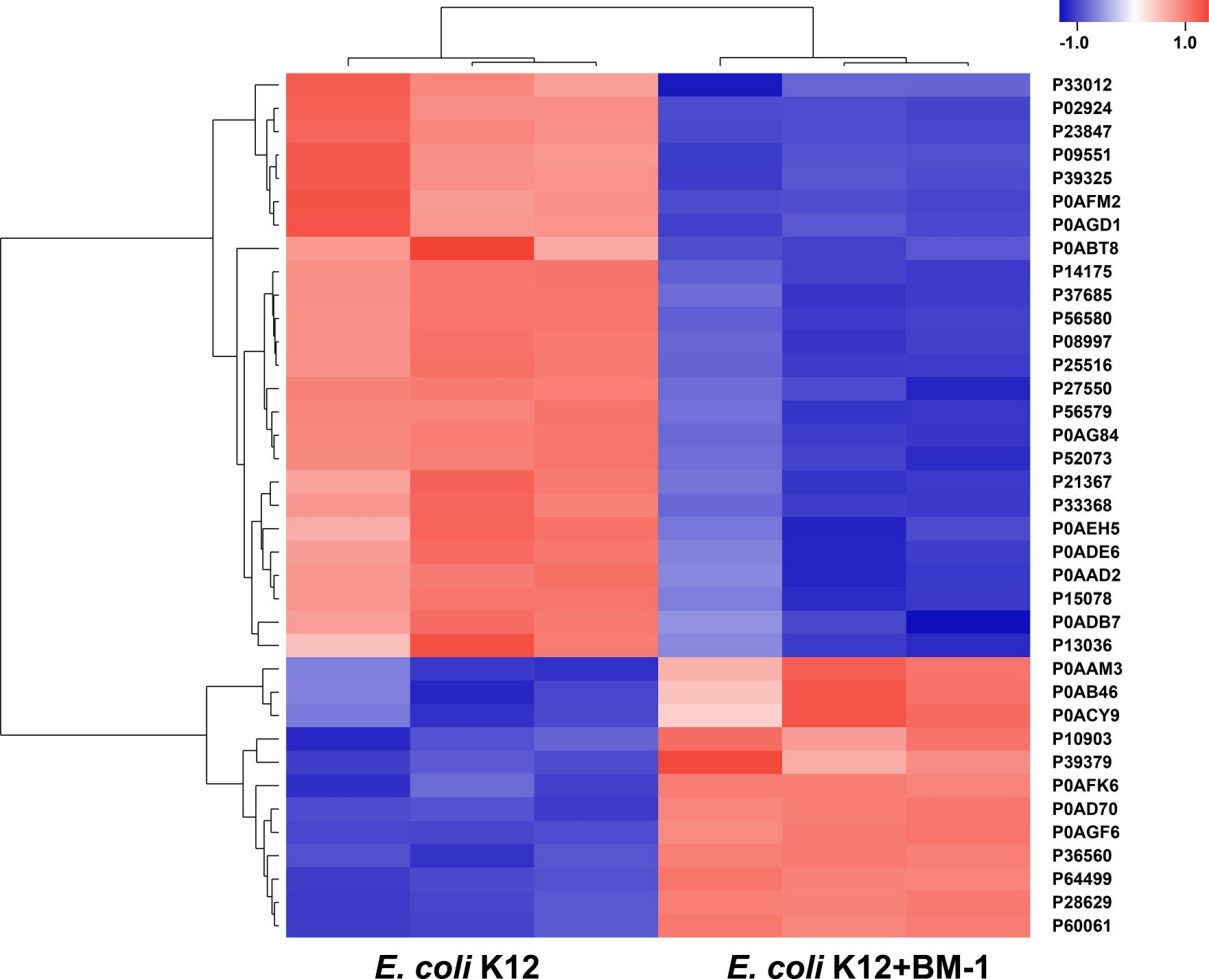
Heat map depicting membrane proteins with a greater than 2-fold change in expression upon *E*. *coli* K12 treatment with plantaricin BM-1. The color in the figure indicates the relative expression level of the proteins; the direction of change is indicated in the color scale.

In addition to peptidoglycan, the membrane structure of cells is also considered to be a barrier against adverse external environments. For example, the antibacterial resistance to nisin is consistently related to the altered fatty acid composition of phospholipids or altered phospholipid composition of the cytoplasmic membrane [28]. Similarly, in this study, 37 of the differentially expressed proteins were membrane proteins and showed more than a 2-fold difference in expression between the control and plantaricin BM-1-treated groups (Fig 5 and S1 Table). The 12 up-regulated membrane proteins mainly comprised transporters and amino acid degradation enzymes, as well as some membrane proteins with unknown function. The (3R)-hydroxymyristol acyl carrier protein dehydratase was up-regulated by 1.37-fold after treatment with plantaricin BM-1 (S1 Table). This enzyme converts-hydroxyacyl-ACPs to trans-2 unsaturated acyl-ACPs [29] and then catalyzes the last step of each fatty acid elongation cycle [30, 31]. Thus, a change in the expression of this enzyme may affect the interaction between plantaricin BM-1 and the target cell membrane, thereby affecting the overall sensitivity of *E*. *coli* K12 to plantaricin BM-1.

Apart from preventing contact between plantaricin BM-1 and the cell membrane, changes of the cell membrane proteins themselves may also affect the sensitivity of *E*. *coli* K12 to plantaricin BM-1. There was no change in the IIC/D of man-PTS, which is the binding target between sensitive gram-positive bacteria and class IIa bacteriocins, indicating that it is not the binding site between *E*. *coli* K12 and plantaricin BM-1 [12]. In addition, the glucitol/sorbitol-specific enzyme IIB/C component of the PTS was found to be significantly down-regulated, and the expression of some transporters also decreased under plantaricin BM-1 exposure. Therefore, further studies should focus on whether the IIB/C component of PTS gluconol/sorbitol- specific enzyme is the binding target between gram-negative bacteria and class IIa bacteriocins.

Moreover, the expression level of the dipeptide transporter DppA was reduced, and the expression of proteins of the dipeptide and oligopeptide transport systems (DppA/C/D/F, OppA/B/C/D/F) was down-regulated by more than 1.2-fold. This finding suggests that the uptake of amino acids in these two systems is related. Such weakening of both oligopeptide and dipeptide transport systems indicates that reduction in amino acid uptake would decrease amino acid metabolism and other physiological activities. Indeed, the expression of the amino acid-binding proteins GltI and LivJ was down-regulated by 1.51- and 1.63-fold, respectively, which can be repressed by the same sRNA antisense regulator (*gcvB*), along with OppA and DppA.

In conclusion, we have demonstrated that plantaricin BM-1 causes cell rupture and depression by interacting with cell membrane, thereby inhibiting the growth of *E*. *coli* K12. A variety of pathways are involved in the response to the stress induced by plantaricin BM-1 in *E*. *coli* K12. Specifically, *E*. *coli* K12 attempts to escape this stress by accelerating peptidoglycan synthesis and the TCA cycle and by regulating the expression of membrane proteins to maintain integrity of the cellular ultrastructure. In contrast to antibiotics, bacteriocin is generally considered more natural [32, 33]. This study thereby lays the foundation for gaining a better understanding of the mechanism of class IIa bacteriocins on gram-negative bacteria, opening the door for new treatment strategies for antibiotic-resistant strains.

## Supporting information

S1 Table More than 2-fold differentially regulated membrane protein of *E*. *coli* K12 under plantaricin BM-1. (DOC)
