# Supplementary material for: Quantitative proteomic analysis reveals the influence of plantaricin BM-1 on metabolic pathways and peptidoglycan synthesis in Escherichia coli K12
Source: PLoS One. 2020 Apr 23;15(4):e0231975. doi: 10.1371/journal.pone.0231975 (PMC7179913; doi:10.1371/journal.pone.0231975)
Supplement: S1 Table — (DOC) [file pone.0231975.s001.doc]

**Table A1:** More than 2-fold differentially regulated membrane protein of *E. coli* K12 under plantaricin BM-1.

| **Accession** | **Description** | **Fold change** | ***P* value** | **Protein** | **Function** |
| --- | --- | --- | --- | --- | --- |
| P28629 | 窗体顶端  Arginine decarboxylase | 5.12 | 0.000002 | AdiA | 窗体顶端  Enzyme; Degradation of small molecules: Amino acids |
| P36560 | 窗体顶端  Acid shock-inducible periplasmic protein | 4.75 | 0.000001 | Asr | 窗体顶端  Phenotype; Not classified |
| P60061 | 窗体顶端  Arginine:agmatine antiporter | 3.92 | 0.000006 | AdiC | 窗体顶端  Putative transport; Not classified |
| P0AD70 | 窗体顶端  窗体顶端  D-alanyl-D-alanine-carboxypeptidase/endopeptidase; penicillin-binding protein; weak beta-lactamase | 2.99 | 0.000004 | AmpH | 窗体顶端  Enzyme; Murein recycling |
| P64499 | Putative inner membrane protein | 2.93 | 0.000005 | YebO | 窗体顶端  Unknown |
| P0AAM3 | 窗体顶端  Hydrogenase maturation protein | 2.91 | 0.002973 | HypC | 窗体顶端  Phenotype; Energy metabolism, carbon: Anaerobic respiration |
| P0AGF6 | 窗体顶端   1. threonine dehydratase, catabolic | 2.90 | 0.00001 | TdcB | 窗体顶端  Enzyme; Degradation of small molecules: Amino acids |
| P39379 | 窗体顶端  Nucleoside recognition pore and gate family putative inner membrane transporter | 2.47 | 0.004104 | YjiH | 窗体顶端  n  Unknown |
| P0AFK6 | Spermidine/putrescine transport system permease protein | 2.43 | 0.00001 | PotC | 窗体顶端  Transport; Transport of small molecules: Amino acids, amines |
| P10903 | 窗体顶端  Nitrate/nitrite transporter | 2.35 | 0.000349 | NarK | 窗体顶端  Transport; Transport of small molecules: Anions |
| P0AB46 | Uncharacterized protein | 2.26 | 0.00528 | YmgD | Unknown |
| P0ACY9 | 窗体顶端  DNA damage-inducible protein regulated by LexA | 2.14 | 0.007453 | YebG | Unknown |
| P0AEH5 | 窗体顶端  DUF883 family protein, putative membrane  anchored ribosome-binding protein | -2.00 | 0.001294 | ElaB | Unknown |
| P14175 | Glycine betaine/proline betaine transport system ATP-binding protein | -2.01 | 0.000036 | ProV | 窗体顶端  Transport; Transport of small molecules: Amino acids, amines |
| P15078 | 窗体顶端  Carbon starvation protein involved in peptide utilization; APC peptide transporter family protein | -2.02 | 0.000194 | CstA | 窗体顶端  Putative transport; Not classified |
| P0ADB7 | 窗体顶端  Entericidin B membrane lipoprotein | -2.02 | 0.000839 | EcnB | Unknown |
| P52073 | 窗体顶端  Glycolate oxidase FAD binding subunit | -2.08 | 0.000035 | GlcE | 窗体顶端  Enzyme; Central intermediary metabolism: Pool, multipurpose conversions |
| P27550 | Acetyl-coenzyme A synthetase | -2.10 | 0.00002 | Acs | 窗体顶端  Enzyme; Fatty acid and phosphatidic acid biosynthesis |
| P25516 | 窗体顶端  Aconitate hydratase 1 | -2.10 | 0.000075 | AcnA | 窗体顶端  Enzyme; Energy metabolism, carbon: TCA cycle |
| P0ABT8 | 窗体顶端  EamA-like transporter family protein | -2.12 | 0.004633 | YijE | Unknown |
| P0AG84 | 窗体顶端  Putative oxidoreductase | -2.12 | 0.000016 | YghA | 窗体顶端  Putative enzyme; Not classified |
| P0AFM2 | 窗体顶端  窗体顶端  High-affinity transport system for glycine betaine and proline | -2.12 | 0.001309 | ProX | 窗体顶端  Transport; Osmotic adaptation |
| P13036 | 窗体顶端  Ferric citrate outer membrane transporter | -2.12 | 0.005351 | FecA | 窗体顶端  Membrane; Transport of small molecules: Cations |
| P33368 | 窗体顶端  Putative oxidoreductase | -2.15 | 0.000221 | YohF | 窗体顶端  Putative enzyme; Not classified |
| P33012 | DNA gyrase inhibitor | -2.20 | 0.001093 | SbmC | Unknown |
| P21367 | 窗体顶端  Putative isochorismatase family hydrolase | -2.27 | 0.00081 | YcaC | Unknown |
| P09551 | 窗体顶端  Lysine/arginine/ornithine transporter subunit | -2.53 | 0.000915 | ArgT | 窗体顶端  Transport; Transport of small molecules: Amino acids, amines |
| P08997 | Malate synthase A | -2.55 | 0.000062 | AceB | 窗体顶端  Enzyme; Central intermediary metabolism: Glyoxylate bypass |
| P0ADE6 | Potassium binding protein | -2.76 | 0.000546 | Kbp | Unknown |
| P23847 | 窗体顶端  Dipeptide transporter | -2.76 | 0.000221 | DppA | 窗体顶端  Transport; Not classified |
| P56580 | 窗体顶端  Glucitol/sorbitol-specific enzyme IIB component of PTS | -2.81 | 0.000052 | SrlE | 窗体顶端  Transport; Transport of small molecules: Carbohydrates, organic acids, alcohols |
| P0AAD2 | Tryptophan-specific transport protein | -2.81 | 0.000303 | Mtr | 窗体顶端  Transport; Transport of small molecules: Amino acids, amines |
| P39325 | 窗体顶端  Galactofuranose binding proteint: periplasmic-binding component of ABC superfamily | -2.92 | 0.00089 | YtfQ | 窗体顶端  Putative regulator; Not classified |
| P37685 | Aldehyde dehydrogenase B | -2.92 | 0.000061 | AldB | 窗体顶端  Enzyme; Degradation of small molecules: Carbon compounds |
| P0AGD1 | 窗体顶端  Superoxide dismutase, Cu, Zn, periplasmic | -3.20 | 0.001704 | SodC | 窗体顶端  Enzyme; Detoxification |
| P02924 | L LLL窗体顶端  L-窗体顶端  L-arabinose transporter subunit | -3.20 | 0.000522 | AraF | 窗体顶端  Transport; Transport of small molecules: Carbohydrates, organic acids, alcohols |
| P56579 | 窗体顶端  Glucitol/sorbitol-specific enzyme IIC component of PTS | -3.24 | 0.000031 | SrlA | 窗体顶端  Transport; Transport of small molecules: Carbohydrates, organic acids, alcohols |
